# Prediction of aortic valve regurgitation after continuous-flow left ventricular assist device implantation using artificial intelligence trained on acoustic spectra

**DOI:** 10.1007/s10047-020-01243-3

**Published:** 2021-02-04

**Authors:** Yusuke Misumi, Shigeru Miyagawa, Daisuke Yoshioka, Satoshi Kainuma, Takuji Kawamura, Ai Kawamura, Yuichi Maruyama, Takayoshi Ueno, Koichi Toda, Hidetsugu Asanoi, Yoshiki Sawa

**Affiliations:** 1 Department of Cardiovascular Surgery, Osaka University Graduate School of Medicine, 2-2-E1, Yamadaoka, Suita City, Osaka 565-0871 Japan; 2 Department of Chronic Heart Failure Management, Osaka University Global Center for Medical Engineering and Informatics, Osaka, Japan; 3 Department of Medical Engineering, Osaka University Hospital, Osaka, Japan

**Keywords:** Aortic valve regurgitation, Left ventricular assist device, Machine learning, Acoustic spectra

## Abstract

Significant aortic regurgitation (AR) is a common complication after continuous-flow left ventricular assist device (LVAD) implantation. Using machine-learning algorithms, this study was designed to examine valuable predictors obtained from LVAD sound and to provide models for identifying AR. During a 2-year follow-up period of 13 patients with Jarvik2000 LVAD, sound signals were serially obtained from the chest wall above the LVAD using an electronic stethoscope for 1 min at 40,000 Hz, and echocardiography was simultaneously performed to confirm the presence of AR. Among the 245 echocardiographic and acoustic data collected, we found 26 episodes of significant AR, which we categorized as “present”; the other 219 episodes were characterized as “none”. Wavelet (time–frequency) analysis was applied to the LVAD sound and 19 feature vectors of instantaneous spectral components were extracted. Important variables for predicting AR were searched using an iterative forward selection method. Seventy-five percent of 245 episodes were randomly assigned as training data and the remaining as test data. Supervised machine learning for predicting concomitant AR involved an ensemble classifier and tenfold stratified cross-validation. Of the 19 features, the most useful variables for predicting concomitant AR were the amplitude of the first harmonic, LVAD rotational speed during intermittent low speed (ILS), and the variation in the amplitude during normal rotation and ILS. The predictive accuracy and area under the curve were 91% and 0.73, respectively. Machine learning, trained on the time–frequency acoustic spectra, provides a novel modality for detecting concomitant AR during follow-up after LVAD.

## Introduction

Left ventricular assist devices (LVADs) have become an important treatment of choice for patients with end-stage heart failure, not only as a bridge to heart transplantation, but also as a destination therapy [1]. With the growing number of patients receiving implantable LVAD therapy, there is an increasing clinical need to sensitively detect LVAD malfunction in the daily lives of patients at home, which could be achieved through use of a tele-monitoring medical care system in the near future. Despite continuing improvements in device design, current LVAD therapies are yet to be free from device-related complications [2, 3]. In patients receiving implantable LVAD therapy, thrombus formation in the LVAD circuit can be implicated by laboratory findings of hemolysis, indicated by elevated levels of serum lactate dehydrogenase (LDH) [4], or significant aortic regurgitation (AR), which can be diagnosed with echocardiography. However, these diagnostic modalities require hospital visits and are not suitable for long-term tele-monitoring at home, especially for LVAD implantation as a destination therapy. Some LVADs have a function allowing detection of device malfunctions, such as detecting pump thrombosis by monitoring power consumption. However, these functions are not sufficiently sensitive to detect slight dysfunctions early enough to prevent the development of serious complications [5, 6]. On the other hand, the LVAD sound is directly derived from the vibration of the device itself and can directly reflect a slight change in the functional status of the device. Attempts have been made to detect LVAD malfunctions by analyzing LVAD sounds, but their diagnostic accuracy has not been satisfactory [7, 8]. This is because the information contained in the LVAD sound is complicated and vast, and it is difficult to analyze it using the commonly used linear statistical analysis methods. Machine learning is a useful technique for detecting complex characteristic patterns from vast datasets [9], and it may be possible to detect LVAD malfunctions by analyzing LVAD sounds.

The purpose of the present study was to determine whether acoustic signal analyses and machine-learning modeling could be used to detect serious AR occurring after LVAD implantation.

## Materials and methods

### Patients

We prospectively followed 13 patients who received the Jarvik2000 continuous-flow implantable LVAD as a bridge to heart transplantation between 2015 and 2017. LVAD was implanted in a standard manner with cardiopulmonary bypass. The pump housing was implanted at the apex of the left ventricle, and the outflow graft was anastomosed to the ascending aorta. The driveline cable passed the subfascial route and exited the right abdominal wall. All patients provided informed consent for participation in this study, and hospital institutional review board approval (No. 15335) was obtained.

### LVAD sound signal collection

Sound signals were obtained on the chest wall just above the LVAD implantation site, with a Bresco commercially available electronic stethoscope (AD Soar, Kanagawa, Japan). Acoustic data were collected monthly at each outpatient visit, or more frequently for inpatients depending on the patients’ clinical status. The Jarvik2000 features intermittent low-speed (ILS) function, which reduces the rotational speed to 7000 revolutions per minute (rpm) for 8 s every 64 s, to allow opening of the aortic valve for the purpose of reducing the risk of thrombus formation by washing-out effect of the native heartbeat [10]. Therefore, each recording lasted for at least 1 min, which included the ILS period. A frequency spectrum between 20 and 2000 Hz was recorded with a sampling rate of 40,000 Hz. Each sound signal was stored in a waveform audio file format.

### Assessment of AR

The grade of AR was confirmed by the transthoracic echocardiography performed at a time closest to the time of the LVAD sound recording. The grade of AR was assessed by the width of the regurgitant jet on the color-Doppler image, divided by the dimension of the aortic annulus, and was categorized as none, trivial, mild, moderate, or severe. In this study, moderate or severe grades of AR were judged as “significant” AR.

### LVAD sound signal processing and feature extraction

The sound signal processing and visualization were performed using custom software developed in MATLAB (version 2018a, MathWorks, Natick, MA). Generally, sound is composed of multiple sound waves of different frequencies (Hertz: fluctuations or unit/time) and amplitudes (sound strength). Spectrographic analysis was performed for each sound signal by plotting acoustic data as a function of time, frequency, and amplitude based on the discrete wavelet transform using a Gabor basis wavelet. Because the amplitude of the recorded sound is susceptible to environmental conditions such as the physical distance between the LVAD and electronic stethoscope, the magnitude of the amplitude fraction was normalized to the percentage of the largest amplitude that was observed within the selected time period (arbitrary unit; a.u.).

The acoustic spectrum from LVAD generally has recognizable peaks produced by the turning of the impeller (e.g., 10,000 rpm corresponds to a 167 Hz peak) and its harmonics. The Jarvik2000, operating normally, rotates at between 8000 and 12,000 rpm, so the basic frequency peak of the impeller appears between 133 and 200 Hz. The second harmonic frequency peak, which is double the basic frequency, appears between 266 and 400 Hz. According to these recognizable peaks, the frequency spectrum in this study was divided into frequency regions to facilitate further calculations (low frequency range: frequency below the basic frequency peak; middle frequency range: between the basic and the second harmonic peak; high frequency range: above the second harmonic peak). The frequency and amplitude fractions of the basic, second, and third harmonic peaks were further processed using a fast Fourier transform. The fluctuations of the frequency and amplitude fractions from each peak were calculated in the order of Hz and a.u., respectively.

While analyzing the frequency fluctuations of the basic sound, a strong peak is usually observed between 1 and 1.5 Hz, which corresponds to the heart rate of the patient. The analysis of amplitude fluctuation also reveals a strong peak below 0.5 Hz, which corresponds to the respiratory rate of the patient.

During the period of ILS operation, the acoustic spectrum from LVAD has the same structural characteristics as the normal operation, which is composed of a recognizable basic peak and its harmonics. The rotational speed at ILS is set at 7,000 rpm, so the basic frequency peak appears around 167 Hz. Fluctuations in the frequency and amplitude fractions of the basic peak during ILS were also analyzed and quantified.

Overall, 19 numerical feature vectors were extracted from each sound signal to prepare for the machine-learning process (Table 1).

**Table 1:** Characteristics of selected features extracted from acoustic spectra obtained from patients with or without aortic regurgitation

	All sound signals				
	Aortic regurgitation (−)		Aortic regurgitation (+)		*p* value
	(N = 219)		(N = 26)		
LVAD operation at normal rotational speed					
Frequency (Hz)					
First harmonics	163 ± 15		165 ± 16		0.74
Second harmonics	326 ± 41		328 ± 32		0.78
Third harmonics	487 ± 34		493 ± 43		0.43
Amplitude of frequency variation (Hz)					
First harmonics	4.2 ± 5.0		3.9 ± 3.8		0.78
Second harmonics	24 ± 22		16 ± 13		0.09
Third harmonics	3.1 ± 9.0		1.5 ± 2.9		0.38
Amplitude of harmonic sounds (a.u.)					
First harmonics	63 ± 15		60 ± 17		0.24
Second harmonics	8.4 ± 9.8		7.9 ± 7.3		0.79
Third harmonics	10 ± 11		8 ± 8		0.86
Amplitude of amplitude variation (a.u.)					
first harmonics	7.1 ± 5.3		7.6 ± 3.4		0.65
Amplitude of non-harmonic sounds (a.u.)					
At low frequency range	6.6 ± 7.8		6.2 ± 2.7		0.77
At middle frequency range	4.4 ± 3.1		3.7 ± 2.2		0.26
At high frequency range	1.2 ± 1.4		0.9 ± 0.9		0.24
LVAD rotational speed (rpm)	10,033 ± 809		10,170 ± 514		0.40
LVAD operation at intermittent low speed					
Amplitude of frequency variation (Hz)					
first harmonics	5.5 ± 4.9		5.6 ± 5.3		0.93
Amplitude of amplitude variation (a.u.)					
first harmonics	3.9 ± 3.5		2.5 ± 2.1		0.05
LVAD rotational speed (rotation per minutes)	7440 ± 904		7072 ± 1001		0.05
Heart rate (bpm)	84 ± 14		79 ± 13		0.09
Respiratory rate (per minute)	20 ± 10		20 ± 6		0.88

Variables are expressed as mean ± standard deviation. Frequency ranges are defined as follows: frequency range lower than the frequency of first harmonics is “low”, range between the first and second harmonics is “middle”, and range higher than the second harmonics is “high”. a.u., arbitrary unit; LVAD, left ventricular assist device

### Machine learning modeling for classifying significant AR

Compared to basic statistical methods and human pattern-recognition abilities, one of the main advantages of machine-learning algorithms is its ability to handle a larger number of features. In contrast to the standard statistical approach, machine learning, which uses automated analytical model building, does not start with a pre-defined model; rather, it allows the data to create the model by detecting underlying patterns while avoiding pre-assumptions regarding model types. The construction of a machine-learning model requires two essential steps: feature selection and the construction of a classification model. A complicated model with many variables is more vulnerable to overfitting. Engineering features that function as foundation for building machine-learning models are quite important to generate high performance of the machine-learning model and for the prevention of overfitting to noise. We performed feature selection with filter and wrapper approaches and then determined the optimal machine-learning algorithm from 8 algorithms. Features were sorted according to their corresponding p value determined by an unpaired t test, and Gini impurity was determined by a random forest built-in feature ranking algorithm. The filter approach was then applied as a pre-processing step by calculating misclassification error with a discrimination analysis while iteratively adding features from the highest rank. Important features were selected when they achieved the minimum misprediction rate. The wrapper approach was applied following the filter approach by performing forward sequential feature selection for the features selected by the filter method to find predictive features and exclude irrelevant features. Misclassification errors were calculated by leave-one-out cross-validation during the feature selection procedure. After all folds were cycled through, the predictions for each fold were aggregated and compared to the true target variable to assess accuracy.

Prediction models for significant AR were developed with 8 machine-learning algorithms, discriminant analysis, k-nearest neighbor (knn: k = 5 with weight), naïve Bayes (NB), kernel NB, random forest, support vector machine (SVM), kernel SVM, and decision tree. We did not use a neural network as a machine-learning algorithm in the current study because of the small sample size. All these algorithms were applied to the best feature combination determined by feature engineering while performing model optimization through hyper-parameter tuning and leave-one-out cross-validation, with the exception of the random forest algorithm. The optimal machine-learning algorithm was determined by comparing its performance. The performance of a built-up machine-learning model was evaluated by misclassification error, area under the curve (AUC), true positive rate, and true negative rate.

## Results

### Patient characteristics

The baseline characteristics of the patients in this study are shown in Table 2. The mean follow-up time was 227 ± 160 days. One patient (Case #2) received concomitant aortic valve replacement with a prosthetic biological valve.

**Table 2:** Patient characteristics

Case number	Age	Sex	Diagnosis	Device	Concomitant procedure	Operation date	Recording period		Recording number		
							From	To	Total, n	AR(+), n	AR(−), n
1	20	Male	DCM	Jarvik2000		2014/6/4	2015/11/12	2015/11/12	1	0	1
2	44	Female	ICM, s/p Nipro-LVAD + TAP	Jarvik2000	AVR(Mosaic Ultra #19)	2016/11/30	2016/12/1	2017/1/30	6	0	6
3	19	Male	DCM, s/p MVR	Jarvik2000		2014/12/20	2015/11/19	2016/11/10	3	0	3
4	44	Female	dHCM	Jarvik2000	Maze, LAA cl	2015/5/15	2015/9/11	2017/4/28	76	0	76
5	56	Male	DCM	Jarvik2000		2016/11/18	2016/11/22	2017/1/25	9	0	9
6	39	Female	DCM	Jarvik2000	TAP	2016/1/13	2016/2/3	2017/4/26	17	10	7
7	36	Female	DCM	Jarvik2000	temporary RVAD	2015/11/11	2015/11/16	2016/6/2	13	0	13
8	59	Female	DCM	Jarvik2000		2016/8/6	2016/8/10	2016/12/1	34	0	34
9	69	Male	Valvular cardiomyopathy	Jarvik2000		2015/9/14	2015/9/25	2016/8/4	13	0	13
10	16	Female	DCM	Jarvik2000	RV plasty, TAP	2015/6/16	2015/9/11	2015/12/24	4	0	4
11	66	Male	dHCM	Jarvik2000		2017/10/6	2017/10/12	2017/10/25	11	0	11
12	39	Female	Puerperal cardiomyopathy	Jarvik2000		2016/3/21	2016/4/18	2017/4/26	16	16	0
13	65	Female	DCM, s/p MVR + CABG	Jarvik2000	Redo MVR(Epic#27)	2016/6/9	2016/6/13	2017/4/26	42	0	42
Total									245	26	219

AR, aortic regurgitation; AVR, aortic valve replacement; CABG, coronary artery bypass grafting; DCM, dilated cardiomyopathy; dHCM, dilated phase of hypertrophic cardiomyopathy; ICM ischemic cardiomyopathy; LAA left atrial appendage; LVAD, left ventricular assist device; MVR, mitral valve replacement; RV, right ventricle; RVAD, right ventricular assist device; TAP, tricuspid annuloplasty

### LVAD sound signal collection and echocardiographic results

**Fig. 1:**
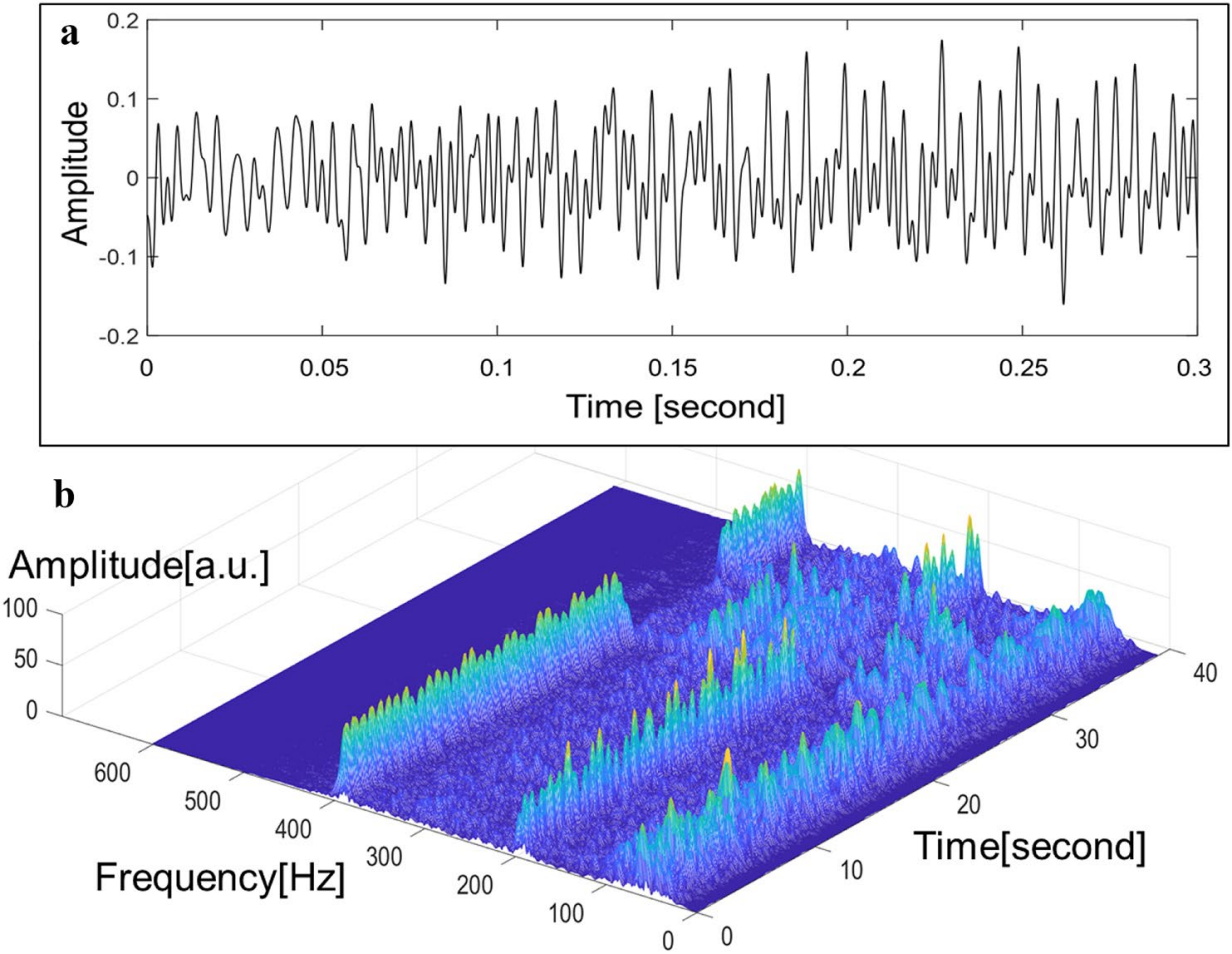
(A) Law sound signals and (B) sound signals processed with the wavelet time–frequency analysis obtained from patients with significant aortic valve regurgitation

**Fig. 2:**
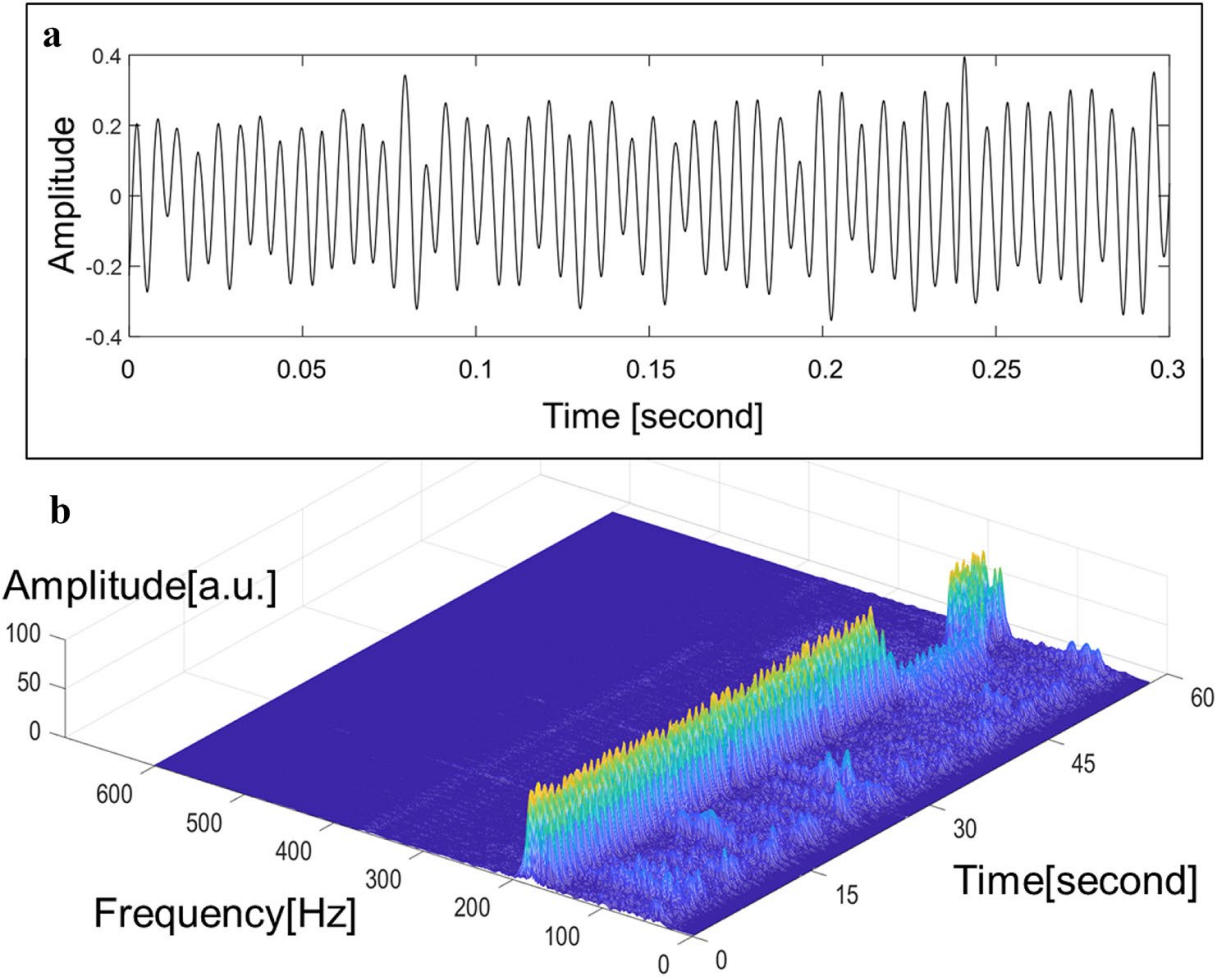
(A) Law sound signals and (B) sound signals processed with the wavelet time–frequency analysis obtained from patients without significant aortic valve regurgitation

During the follow-up period, a total of 245 LVAD sound signals were obtained. Of those, 26 (10.6%) sound signals were obtained from 2 (15%) patients with significant AR (Fig. 1). The remaining 219 (89.4%) sound signals were obtained from patients without significant AR (Fig. 2).

### Feature extraction and selection

**Fig. 3:**
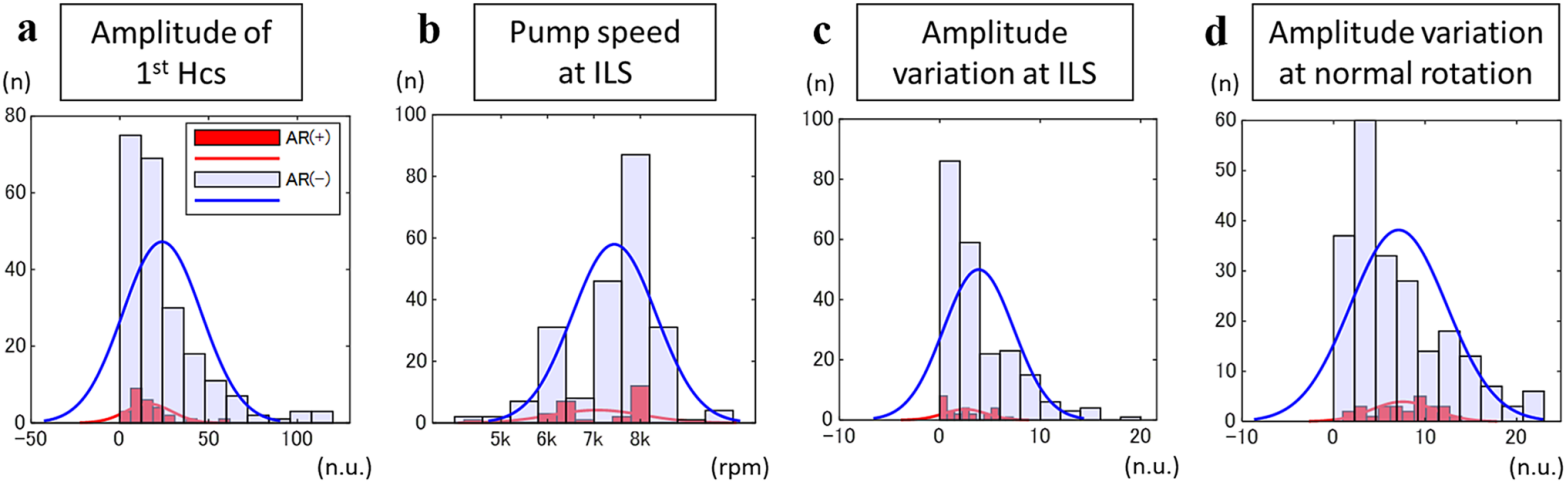
The distributions of the four sets of important features to predict significant aortic regurgitation by sound signals obtained at the timing with and without significant aortic regurgitation: (A) the amplitude of the first harmonics, (B) the LVAD rotational speed, (C) the variation of the amplitude during ILS, and (D) the variation of the amplitude during normal rotation. HCS, harmonics; LVAD, left ventricular assist device; ILS, intermittent low speed

The results and data quality of the extracted features from each sound signal are summarized in Table 1. Utilizing three feature selection techniques, the four important features obtained and their distribution by those with and without AR are shown in Fig. 3. The ensemble classifier and t test identified the following three features as important features for predicting AR: the amplitude of first harmonics, LVAD rotational speed during ILS, and the variation of the amplitude during ILS. In addition, the variation of the amplitude during normal rotation was proposed to also be predictive of AR by sequential feature selection and neighborhood component analysis.

### Selecting the best predictive dataset and classifier algorithm

Utilizing the dataset of important features selected, the ensemble classifier best predicted AR with an AUC of 0.73 (Table 3). The total accuracy of the classifier to distinguish sound data with and without significant AR was 91%, with a true positive rate of 75% to detect significant AR.

**Table 3:** Performance of machine-learning algorithms to detect significant aortic regurgitation

Classifier	Accuracy	Area-under-the-curve	True positive rate	True negative rate
Ensemble	0.91	0.73	0.75	0.92
Random forest	0.91	0.72	0.71	0.91
k-nearest neighbor	0.89	0.55	0.00	0.89
Support vector machine	0.89	0.50	0.11	0.00
Kernel support vector machine	0.89	0.50	0.11	0.00
Naïve Bayes	0.89	0.73	0.50	0.90
Karnel naïve Bayes	0.89	0.73	0.50	0.90
Decision tree	0.89	0.76	0.50	0.94

## Discussion

The major findings of this study can be summarized as follows: in patients with a Jarvik2000 LVAD, the machine-learning algorithm, trained on acoustic spectra obtained from LVAD sound signals, effectively predicted significant AR. During processing of the recorded sound signals, 19 features were extracted from each sound signal using wavelet time–frequency analysis. Of these 19 features, 4 features were selected as important predictors for AR. With this dataset, the ML algorithm was trained and predicted AR with an accuracy of 91%. The observed efficacy suggests that machine learning trained on acoustic spectra has an important role in detecting clinically important conditions in patients with LVAD.

AR is one of the major complications after CF-LVAD implantation, and it is said that mild-to-moderate AR occurs in approximately 25% of cases within one year after surgery [11, 12]. Exacerbation of de novo AR after LVAD implantation leads to heart failure, so it is necessary to detect significant AR at an early stage, so that appropriate therapeutic interventions can be performed, including surgical treatment. De novo AR occurs at a constant rate regardless of the LVAD model [13], and monitoring of AR is still important, despite the continuing improvement in LVAD device design. De novo AR is considered to originate from degeneration of the aortic valve tissue and left ventricular unloading by LVAD, causing a pressure gradient in the opposite direction to the aortic valve [11, 14, 15], both of which are largely unrelated to the improvement of LVAD device design. In this study, we set the detection of significant AR as an outcome and made good predictions using machine-learning modeling. AR is usually followed by echocardiography, but it is not realistic to perform frequent echo examinations in the setting of LVAD destination therapy managed at home. A commercially available electronic stethoscope was used to collect the LVAD sound in this study, demonstrating that it is possible to make a diagnosis during home management of LVAD, which is an advantageous feature in view of LVAD remote management. The acoustic analysis used in this study could be a useful modality for tracking the time course of AR in individual patients at home as well as for the early detection of other LVAD complications such as pump thrombosis.

In utilizing a machine-learning algorithm for predicting the outcome, preparing datasets that are relevant and not redundant is important to achieve satisfactory predictive accuracy. In this study, wavelet time–frequency analysis extracted 19 features characterizing the recognizable peaks of basic rotational sound and its harmonics, and the noises existing between the peaks for each sound signal. This feature extraction might contribute to improving the predictive accuracy of the machine-learning process. To date, acoustic signal analysis has been used to detect pump thrombi but fails to provide accurate diagnostic tools [7, 16–19]. The reason is that current methods of analyzing acoustic signals rely mainly on the distribution of power spectral density, which cannot discriminate between normal and abnormal patterns quantitatively. To enhance the diagnostic capability of acoustic signals, we have to choose methods that adapt to more complex system analyses. The extracted features utilized in this study achieved a more detailed characterization of each LVAD sound signal, which led to more accurate detection of concomitant clinical settings.

Feature selection is also an important step in achieving improved predictive accuracy in the machine-learning process. Choosing a set of features that has the best predictive value and eliminating the redundancy between extracted features is useful not only for enhancing the predictive accuracy of machine learning but also for reducing the number of calculations the algorithm has to process. Previous machine-learning articles have utilized several different algorithms at the feature selection step [20–22]. There is no single algorithm to detect important features to predict the outcome. In this study, we utilized three different ways of selecting important features and compared them to determine the best set of features with a high predictive value. This process might also contribute to achieving the high predictive value of the following machine-learning process.

Feature selection is also beneficial for clinicians because the selected important features enable us to interpret the relationships between the features and the outcome. In this study, three features were selected as being highly predictive of concomitant AR: the amplitude of the first harmonic, LVAD rotational speed during ILS, and the variation of the amplitude both during normal rotation and ILS. The first harmonic is the most basic sound produced by the LVAD rotation, reflecting the rotational speed of the device. In cases without significant AR, these first harmonics appear prominently, while in cases with AR, acoustic components appear in addition to the first harmonics; therefore, it is presumed that the intensity of the first harmonics are relatively weakened. In AR cases, the LVAD rotational speed tended to decrease more during ILS than in non-AR cases. In AR cases, the LVAD preload increases with AR, and it is presumed that this works in the direction of lowering the LVAD rotational speed to a greater extent than in non-AR cases. Furthermore, in AR cases, the intensity of the LVAD sound fluctuated to a greater degree. The fluctuation range of the intensity of the LVAD sound seems to represent the change in the amount of work performed by the LVAD, and this may depend on the change in the preload of the LVAD. In AR cases, as a result of insufficient unloading of the left ventricle due to AR, it is presumed that the increase and/or decrease in preload of the LVAD, caused by the native cardiac output, appears more easily than in non-AR cases, which affects the degree of fluctuation of the device’s rotational speed. Further research is needed with more large number of clinical datasets or bench testing with artificial mock circulation to clarify the clinical implications of these important predictive features for the detection of AR.

Among the three important features selected to predict concomitant AR included acoustic data during ILS. As ILS is a function specifically equipped with Jarvik2000, our results cannot be simply applied to other LVAD systems. However, methodologies described in this study including feature extraction from acoustic signals, selecting the best predictive dataset, and choosing best classifier algorithm to predict clinical outcome, can be theoretically applied to other LVAD systems with clinical outcomes other than AR.

## Conclusion

Machine learning trained on the time–frequency acoustic spectra provides a novel modality for detecting concomitant AR during follow-up after CF-LVAD implantation. This could allow automated identification of pathological conditions related to the device, resulting in earlier diagnosis and subsequent intervention.
